# The SpineML toolchain: enabling computational neuroscience through flexible tools for creating, sharing, and simulating neural models

**DOI:** 10.1186/1471-2202-15-S1-P224

**Published:** 2014-07-21

**Authors:** Alexander J Cope, Paul Richmond, Dave Allerton

**Affiliations:** 1 Department of Computer Science, University of Sheffield, Sheffield, South Yorkshire, S10 2TN, UK; 2 Department of ACSE, University of Sheffield, Sheffield, South Yorkshire, S10 2TN, UK

## 

Within computational neuroscience the convergence of large scale computation and biology offers the potential to offer new insight into biological function. Conversely, the simulation of large scale biological systems has the potential to revolutionise computing systems and software which are increasingly showing a trend towards lower power and increased parallelism. In order to achieve large scale simulation, computational neuroscience projects must increase in both scale and complexity. With these increases the problems of managing computational models, sharing these models, and simulating the models rapidly and efficiently become increasingly important to the rate of scientific progress. We present a flexible toolchain that seeks to tackle these issues by providing a standardised pipeline for the specification and simulation of large scale neural models (consisting of rate-coded or point neurons).

As the basis of this toolchain we have developed SpineML [1], an XML standard for model description. SpineML ensures compatibility between different models as it contains no simulator or authoring tool specific implementation details, allowing models developed using different tools with different simulators to be combined without alteration. This allows collaborators to develop models independently, using SpineML as a common interchange format.

Models authored in SpineML can easily be used in a ‘systems integration’ approach to computational neuroscience. Numerous models can be combined as system components to form models of increasing complexity. This approach can be used to accelerate the progress of research by avoiding the recreation of existing models. SpineML also facilitates model sharing through layer oriented design [2] which provides clear separation of experimental details. This allows changes to simulation parameters, inputs and outputs, and model properties to be performed in an experiment file without changing the model itself and allows collaborators to test different conditions without making changes to the shared model.

SpineML provides syntax governed by clearly specified XML schemas. This ensures compatibility between models, however does not place any restriction is on the tools used for authoring models. We have produced a reference model creation tool, SpineCreator, which provides a graphical interface to the SpineML format. This tool allows SpineML models to be created using graphical methods, and visualized in 3D, and is designed to require no knowledge of programming to use, thus making the toolchain accessible to the widest range of users possible.

Simulators can support SpineML network descriptions at two levels, with the lowest level interface allowing greater flexibility of the specification and connectivity of the neuronal dynamics. Multi-level support allows SpineML models to be supported and deployed on a wide range of simulators and simulator hardware. Simulation support is currently available using a range of traditional simulators (via PyNN), a reference simulator (BRAHMS) and simulation support for GPUs via GeNN is under development. In addition to this a toolchain for the low power, highly distributed, SpiNNaker hardware architecture has been developed. SpineML facilitates the accessibility of this neuromorphic platform by providing a modeling framework which can be used to develop and test models using traditional simulators which can then be deployed without change directly to neuromorphic hardware.

This work was funded by the EPSRC (BIMPA Project (EP/G015740/1) and Green Brain Project (EP/J019690/1)).
